# Supplementary figures and images for: Marek’s disease virus prolongs survival of primary chicken B-cells by inducing a senescence-like phenotype
Source: PLoS Pathog. 2021 Oct 21;17(10):e1010006. doi: 10.1371/journal.ppat.1010006 (PMC8562793; doi:10.1371/journal.ppat.1010006)

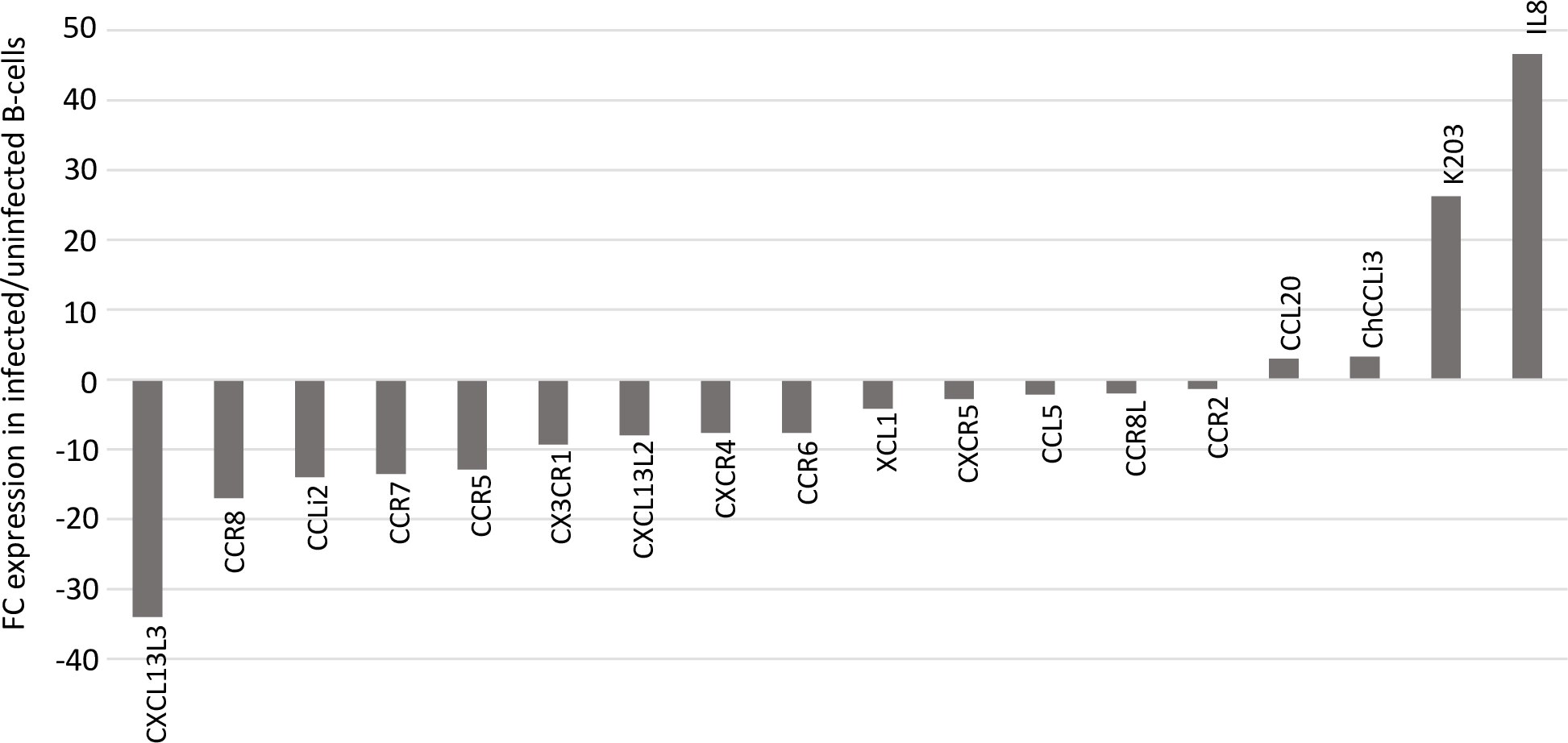

Supplement: S1 Fig — Microarray analysis revealed that 18 chemokines/chemokines receptors genes involved in cytokine/cytokine receptors interaction pathways are differentially expressed in RB1B-GFP-infected B-cells. Results are presented as fold change (FC) for the mRNA expression of each gene in MDV-infected B-cells relative to uninfected B-cells. (TIF) [file ppat.1010006.s001.tif]
