# Supplementary material for: Marek’s disease virus prolongs survival of primary chicken B-cells by inducing a senescence-like phenotype
Source: PLoS Pathog. 2021 Oct 21;17(10):e1010006. doi: 10.1371/journal.ppat.1010006 (PMC8562793; doi:10.1371/journal.ppat.1010006)
Supplement: S2 Table — (DOCX) [file ppat.1010006.s003.docx]

| Gene Symbol | Gene description | FC | P value |
| --- | --- | --- | --- |
| CXCL13L3 | C-X-C motif chemokine ligand 13-like 3 precursor | -34 | 0,00E+00 |
| IL18 | interleukin 18 | -18 | 0,00E+00 |
| CCR8 | C-C motif chemokine receptor 8 | -17 | 0,00E+00 |
| IL2RA | IL2RA interleukin 2 receptor subunit alpha | -15 | 0,00E+00 |
| CCLi2 | C-C motif chemokine 4 homolog precursor | -14 | 1,00E-06 |
| CCR7 | chemokine (C-C motif) receptor 7 | -14 | 0,00E+00 |
| TNFRSF11B | TNF receptor superfamily member 11b | -13 | 1,00E-06 |
| CCR5 | C-C motif chemokine receptor 5 | -13 | 0,00E+00 |
| IL21R | interleukin 21 receptor | -11 | 0,00E+00 |
| CX3CR1 | C-X3-C motif chemokine receptor 1 | -9 | 0,00E+00 |
| IL7R | interleukin 7 receptor | -9 | 1,00E-06 |
| CXCL13L2 | C-X-C motif chemokine ligand 13-like 2 precursor | -8 | 0,00E+00 |
| IFNG | interferon, gamma | -8 | 0,00E+00 |
| CXCR4 | chemokine (C-X-C motif) receptor 4 | -8 | 0,00E+00 |
| CCR6 | chemokine (C-C motif) receptor 6 | -8 | 0,00E+00 |
| TNFRSF13B | tumor necrosis factor receptor superfamily, member 13B | -7 | 0,00E+00 |
| CSF2RA | granulocyte-macrophage colony-stimulating factor receptor subunit alpha-like | -6 | 1,90E-05 |
| XCL1 | X-C motif chemokine ligand 1 precursor | -4 | 3,86E-02 |
| GDF9 | growth/differentiation factor 9 | -4 | 4,82E-04 |
| IL28RA | interleukin 28 receptor, alpha (interferon, lambda receptor) | -4 | 1,00E-06 |
| FAS | Fas cell surface death receptor | -4 | 4,90E-05 |
| CD40 | CD40 molecule, TNF receptor superfamily member 5 | -4 | 2,80E-05 |
| TNFSF13B | tumor necrosis factor (ligand) superfamily, member 13b | -3 | 1,84E-03 |
| IL20RA | interleukin 20 receptor, alpha | -3 | 4,80E-05 |
| IL2RG | interleukin 2 receptor subunit gamma | -3 | 2,30E-05 |
| TGFBR2 | transforming growth factor, beta receptor II (70/80kDa) | -3 | 1,30E-05 |
| TGFB1 | transforming growth factor beta 1 | -3 | 8,10E-04 |
| CXCR5 | chemokine (C-X-C motif) receptor 5 | -3 | 7,02E-04 |
| GHR | growth hormone receptor | -3 | 7,11E-03 |
| IL17RD | interleukin 17 receptor D | -3 | 1,03E-02 |
| IL6ST | interleukin 6 signal transducer (gp130, oncostatin M receptor) | -2 | 5,74E-03 |
| TNFRSF9 | tumor necrosis factor receptor superfamily member 9 | -2 | 2,20E-04 |
| CCL5 | chemokine (C-C motif) ligand 5 | -2 | 3,86E-03 |
| TGFB3 | transforming growth factor beta 3 | -2 | 1,33E-04 |
| IL16 | interleukin 16 | -2 | 7,09E-04 |
| CCR8L | C-C chemokine receptor 8 like | -2 | 1,75E-03 |
| IL13RA2 | interleukin 13 receptor, alpha 2 | -2 | 1,53E-04 |
| IL2 | interleukin 2 precursor | -2 | 6,24E-03 |
| TNFRSF8 | tumor necrosis factor receptor superfamily, member 8 | -2 | 8,36E-02 |
| CCR2 | chemokine (C-C motif) receptor 2 | -1 | 1,64E-01 |
| CSF2RB | colony stimulating factor 2 receptor,beta,low-affinity (granulocyte-macrophage) | -1 | 5,90E-01 |
| IFNB | interferon beta | 2 | 1,18E-03 |
| IL15RA | interleukin-15 receptor subunit alpha isoform X2 | 2 | 2,10E-05 |
| IL10RB | interleukin 10 receptor, beta | 2 | 9,78E-04 |
| CCL20 | C-C motif chemokine 20 precursor | 3 | 6,34E-04 |
| TNFSF15 | tumor necrosis factor (ligand) superfamily, member 15 | 3 | 2,32E-04 |
| IL6 | interleukin 6 | 3 | 1,45E-04 |
| chCCLi3 | chCCLi3/ K203 | 3 | 7,65E-03 |
| K203 | chemokine (C-C motif) ligand 4 | 26 | 0,00E+00 |
| IL8 | interleukin 8 = CXCLi2 | 47 | 0,00E+00 |
