# Supplementary material for: Marek’s disease virus prolongs survival of primary chicken B-cells by inducing a senescence-like phenotype
Source: PLoS Pathog. 2021 Oct 21;17(10):e1010006. doi: 10.1371/journal.ppat.1010006 (PMC8562793; doi:10.1371/journal.ppat.1010006)
Supplement: S3 Table — (DOCX) [file ppat.1010006.s004.docx]

| Gene Symbol | Gene description | FC | P value |
| --- | --- | --- | --- |
| MYC | v-myc avian myelocytomatosis viral oncogene homolog | -9 | 0,00E+00 |
| CCNA1 | cyclin A1 | -8 | 9,00E-06 |
| CCNE2 | G1/S-specific cyclin-E2 | -7 | 0,00E+00 |
| ORC1 | origin recognition complex subunit 1 | -5 | 1,00E-06 |
| CDC20 | cell division cycle 20 | -5 | 0,00E+00 |
| MCM3 | minichromosome maintenance complex component 3 | -4 | 3,00E-06 |
| CCND1 | G1/S-specific cyclin-D1 | -4 | 1,26E-04 |
| MCM5 | minichromosome maintenance complex component 5 | -4 | 1,90E-05 |
| CCND3 | G1/S-specific cyclin-D3 | -4 | 1,00E-06 |
| CDK1 | cyclin dependent kinase 1 | -4 | 2,00E-06 |
| BUB1 | BUB1 mitotic checkpoint serine/threonine kinase | -4 | 1,74E-04 |
| CCNA2 | cyclin A2 | -4 | 9,00E-06 |
| PLK1 | polo-like kinase 1 | -4 | 4,40E-05 |
| CHEK1 | serine/threonine-protein kinase Chk1 | -4 | 7,00E-06 |
| PCNA | proliferating cell nuclear antigen | -3 | 2,00E-06 |
| CCNB2 | G2/mitotic-specific cyclin-B2 | -3 | 2,10E-05 |
| BUB1B | BUB1 mitotic checkpoint serine/threonine kinase B | -3 | 1,80E-05 |
| CDC45 | cell division cycle 45 | -3 | 1,59E-03 |
| CDKN2A | cyclin-dependent kinase inhibitor 2A (melanoma, p16, inhibits CDK4) | -3 | 1,19E-02 |
| MCM6 | minichromosome maintenance complex component 6 | -3 | 7,54E-04 |
| WEE1 | WEE1 G2 checkpoint kinase | -3 | 1,90E-05 |
| MCM2 | minichromosome maintenance complex component 2 | -3 | 6,00E-06 |
| TGFB1 | transforming growth factor beta 1 | -3 | 8,10E-04 |
| E2F1 | E2F transcription factor 1 | -3 | 5,00E-05 |
| ORC5 | origin recognition complex, subunit 5 | -3 | 1,40E-05 |
| PTTG1 | pituitary tumor-transforming 1 | -3 | 2,70E-05 |
| CDC14B | cell division cycle 14B | -2 | 2,00E-03 |
| CCNB3 | G2/mitotic-specific cyclin-B3 | -2 | 2,07E-04 |
| RBL1 | RB transcriptional corepressor like 1 | -2 | 4,08E-04 |
| CCNI | cyclin I | -2 | 2,85E-03 |
| TTK | TTK protein kinase | -2 | 4,99E-03 |
| CDC7 | cell division cycle 7 | -2 | 1,86E-03 |
| E2F5 | E2F transcription factor 5, p130-binding | -2 | 6,13E-04 |
| ORC2 | origin recognition complex subunit 2 | -2 | 1,78E-04 |
| TGFB3 | transforming growth factor beta 3 | -2 | 1,33E-04 |
| ANAPC2 | anaphase promoting complex subunit 2 | -2 | 5,35E-03 |
| ORC3 | origin recognition complex subunit 3 | -2 | 5,52E-02 |
| ANAPC10 | anaphase promoting complex subunit 10 | -2 | 4,31E-02 |
| GADD45B | growth arrest and DNA-damage-inducible, beta | 2 | 8,62E-02 |
| RB1 | retinoblastoma 1 | 2 | 1,27E-03 |
| CDKN1B | cyclin-dependent kinase inhibitor 1B (p27, Kip1) | 3 | 1,09E-03 |
| GADD45A | growth arrest and DNA-damage-inducible, alpha | 4 | 3,00E-05 |
| CIP1 | cdk inhibitor CIP1 (p21) | 5 | 0,00E+00 |
| CDKN2B | cyclin-dependent kinase inhibitor 2B (melanoma, p16, inhibits CDK4) | 7 | 4,96E-04 |
