# Supplementary material for: Marek’s disease virus prolongs survival of primary chicken B-cells by inducing a senescence-like phenotype
Source: PLoS Pathog. 2021 Oct 21;17(10):e1010006. doi: 10.1371/journal.ppat.1010006 (PMC8562793; doi:10.1371/journal.ppat.1010006)
Supplement: S4 Table — (DOCX) [file ppat.1010006.s005.docx]

| Gene Symbol | Gene description | FC | P value |
| --- | --- | --- | --- |
| BCL2A1 | BCL2-related protein A1 | -7 | 0,00E+00 |
| CTSC | dipeptidyl peptidase 1 precursor | -7 | 0,00E+00 |
| BCL2L1 | BCL2-like 1 | -6 | 0,00E+00 |
| MCL1 | myeloid cell leukemia sequence 1 (BCL2-related) | -5 | 0,00E+00 |
| ATF4 | activating transcription factor 4 (tax-responsive enhancer element B67) | -4 | 0,00E+00 |
| FAS | Fas cell surface death receptor | -4 | 4,90E-05 |
| BCL2 | B-cell CLL/lymphoma 2 | -4 | 7,70E-05 |
| ACTB | actin, cytoplasmic 1 | -4 | 2,20E-05 |
| BIRC5 | survivin isoform 1 | -3 | 3,80E-05 |
| TRAF1 | TNF receptor-associated factor 1 isoform X4 | -3 | 1,61E-04 |
| LMNB2 | lamin-B2 | -3 | 2,39E-03 |
| CTSZ | cathepsin Z | -3 | 1,40E-05 |
| PIK3R1 | phosphoinositide-3-kinase, regulatory subunit 1 (p85 alpha) | -3 | 6,70E-05 |
| TRAF2 | TNF receptor associated factor 2 | -3 | 7,75E-04 |
| NFKB1 | nuclear factor kappa B subunit 1 | -3 | 5,00E-05 |
| ENDOG | endonuclease G | -3 | 2,83E-03 |
| PIK3CD | phosphoinositide-3-kinase, catalytic, delta polypeptide | -2 | 4,72E-04 |
| BID | BH3 interacting domain death agonist | -2 | 1,08E-04 |
| CASP8 | caspase 8, apoptosis-related cysteine peptidase | -2 | 1,83E-03 |
| CFLAR | CASP8 and FADD-like apoptosis regulator | -2 | 1,35E-04 |
| ITPR2 | inositol 1,4,5-trisphosphate receptor type 2 isoform X1 | -2 | 7,41E-03 |
| BAK1 | bcl-2 homologous antagonist/killer | -2 | 3,58E-03 |
| CASP2 | caspase-2 | -2 | 5,17E-04 |
| TUBA3E | tubulin alpha-8 chain | -2 | 4,20E-03 |
| CASP3 | caspase 3, apoptosis-related cysteine peptidase | -1 | 8,43E-02 |
| CSF2RB | colony stimulating factor 2 receptor, beta, low-affinity (granulocyte-macrophage) | -1 | 5,90E-01 |
| GADD45B | growth arrest and DNA damage-inducible protein GADD45 beta | 2 | 8,62E-02 |
| APAF1 | apoptotic peptidase activating factor 1 | 2 | 7,65E-02 |
| IKBKB | inhibitor of kappa light polypeptide gene enhancer in B-cells, kinase beta | 2 | 2,08E-04 |
| CTSD | cathepsin D precursor | 2 | 7,24E-03 |
| MAPK8 | mitogen-activated protein kinase 8 | 2 | 1,70E-03 |
| GADD45A | growth arrest and DNA damage-inducible protein GADD45 alpha | 4 | 3,00E-05 |
