# Supplementary material for: Marek’s disease virus prolongs survival of primary chicken B-cells by inducing a senescence-like phenotype
Source: PLoS Pathog. 2021 Oct 21;17(10):e1010006. doi: 10.1371/journal.ppat.1010006 (PMC8562793; doi:10.1371/journal.ppat.1010006)
Supplement: S5 Table — (DOCX) [file ppat.1010006.s006.docx]

| Gene symbol | | Gene Description | FC | P value |
| --- | --- | --- | --- | --- |
| BCL2L1 | BCL2-like 1 | | -6 | 0,00E+00 |
| RAB8A | RAB8A, member RAS oncogene family | | -5 | 2,00E-06 |
| BCL2 | B-cell CLL/lymphoma 2 | | -4 | 7,70E-05 |
| PRKCD | protein kinase C, delta | | -4 | 7,70E-05 |
| HMGB1 | high-mobility group box 1 | | -4 | 0,00E+00 |
| RHEB | Ras homolog enriched in brain | | -3 | 2,00E-06 |
| PIK3R1 | phosphoinositide-3-kinase, regulatory subunit 1 (p85 alpha) | | -3 | 6,70E-05 |
| PIK3CD | phosphoinositide-3-kinase, catalytic, delta polypeptide | | -2 | 4,72E-04 |
| CFLAR | CASP8 and FADD-like apoptosis regulator | | -2 | 1,35E-04 |
| TRAF6 | TNF receptor-associated factor 6 | | 2 | 1,31E-02 |
| ATG2A | autophagy related 2A | | 2 | 6,14E-03 |
| BNIP3 | BCL2/adenovirus E1B 19kDa interacting protein 3 | | 2 | 1,08E-03 |
| GABARAPL2 | GABA(A) receptor-associated protein-like 2 | | 2 | 3,87E-04 |
| LAMP1 | lysosomal-associated membrane protein 1 | | 2 | 7,45E-04 |
| SQSTM1 | sequestosome 1 | | 2 | 4,43E-03 |
| CTSD | cathepsin D | | 2 | 7,24E-03 |
| MAPK8 | mitogen-activated protein kinase 8 | | 2 | 1,70E-03 |
| RAB33B | RAB33B, member RAS oncogene family | | 3 | 8,00E-06 |
| WIPI1 | WD repeat domain, phosphoinositide interacting 1 | | 4 | 3,00E-06 |
| IGF1R | insulin-like growth factor 1 receptor | | 4 | 5,52E-04 |
| DEPDC6 | DEP domain containing 6 | | 16 | 0,00E+00 |
