# Supplementary material for: Marek’s disease virus prolongs survival of primary chicken B-cells by inducing a senescence-like phenotype
Source: PLoS Pathog. 2021 Oct 21;17(10):e1010006. doi: 10.1371/journal.ppat.1010006 (PMC8562793; doi:10.1371/journal.ppat.1010006)
Supplement: S6 Table — (DOCX) [file ppat.1010006.s007.docx]

| Gene symbol | Gene Description | FC | P value |
| --- | --- | --- | --- |
| MYC | v-myc avian myelocytomatosis viral oncogene homolog | -9 | 0,00E+00 |
| CCNA1 | cyclin-A1 | -8 | 9,00E-06 |
| CCNE2 | G1/S-specific cyclin-E2 | -7 | 0,00E+00 |
| CCND1 | G1/S-specific cyclin-D1 | -4 | 1,26E-04 |
| CCND3 | G1/S-specific cyclin-D3 | -4 | 1,00E-06 |
| CDK1 | cyclin-dependent kinase 1 | -4 | 2,00E-06 |
| RASSF5 | ras association domain-containing protein 5 | -4 | 1,04E-04 |
| ZFP36L1 | mRNA decay activator protein ZFP36L1 | -4 | 4,20E-05 |
| CCNA2 | cyclin-A2 | -4 | 9,00E-06 |
| NFATC1 | nuclear factor of activated T-cells, cytoplasmic 1 isoform X9 | -4 | 1,00E-06 |
| CHEK1 | serine/threonine-protein kinase Chk1 | -4 | 7,00E-06 |
| MYBL2 | myb-related protein B | -3 | 3,00E-06 |
| RHEB | GTP-binding protein Rheb isoform X1 | -3 | 2,00E-06 |
| CCNB2 | G2/mitotic-specific cyclin-B2 | -3 | 2,10E-05 |
| NBN | nibrin | -3 | 6,60E-05 |
| TRAF3IP2 | adapter protein CIKS isoform X4 | -3 | 0,00E+00 |
| VDAC3 | voltage-dependent anion-selective channel protein 3 isoform X2 | -3 | 5,00E-06 |
| TGFBR2 | TGF-beta receptor type-2 precursor | -3 | 1,30E-05 |
| TGFB1 | transforming growth factor beta 1 | -3 | 8,10E-04 |
| PIK3R1 | phosphatidylinositol 3-kinase regulatory subunit alpha isoform X2 | -3 | 6,70E-05 |
| E2F1 | E2F transcription factor 1 | -3 | 5,00E-05 |
| FOXM1 | forkhead box protein M1 | -3 | 1,32E-03 |
| NFKB1 | nuclear factor NF-kappa-B p105 subunit | -3 | 5,00E-05 |
| CCNB3 | G2/mitotic-specific cyclin-B3 | -2 | 2,07E-04 |
| PIK3CD | phosphatidylinositol 4,5-bisphosphate 3-kinase catalytic subunit delta isoform | -2 | 4,72E-04 |
| RBL1 | RB transcriptional corepressor like 1 | -2 | 4,08E-04 |
| HIPK2 | homeodomain-interacting protein kinase 2 isoform X6 | -2 | 1,61E-02 |
| E2F5 | E2F transcription factor 5, p130-binding | -2 | 6,13E-04 |
| ITPR2 | inositol 1,4,5-trisphosphate receptor type 2 isoform X1 | -2 | 7,41E-03 |
| MAP2K3 | dual specificity mitogen-activated protein kinase kinase 3 | -2 | 3,70E-03 |
| PPP1CC | serine/threonine-protein phosphatase PP1-gamma catalytic subunit | -2 | 5,10E-05 |
| TGFB3 | transforming growth factor beta-3 proprotein preproprotein | -2 | 1,33E-04 |
| SLC25A5 | ADP/ATP translocase 2 | -2 | 4,79E-03 |
| FOXO1 | forkhead box protein O1 | -2 | 8,86E-04 |
| GADD45B | growth arrest and DNA damage-inducible protein GADD45 beta | 2 | 8,62E-02 |
| RB1 | retinoblastoma-associated protein | 2 | 1,27E-03 |
| SQSTM1 | sequestosome-1 isoform X1 | 2 | 4,43E-03 |
| FOXO3 | forkhead box protein O3 | 3 | 0,00E+00 |
| IL6 | interleukin-6 precursor | 3 | 1,45E-04 |
| GADD45A | growth arrest and DNA damage-inducible protein GADD45 alpha | 4 | 3,00E-05 |
| CIP1 | cdk inhibitor CIP1 (p21) | 5 | 0,00E+00 |
| VDAC1 | voltage-dependent anion-selective channel protein 1 | 6 | 1,80E-05 |
| CDKN2B | cyclin-dependent kinase inhibitor 2B (melanoma, p16, inhibits CDK4) | 7 | 4,96E-04 |
