# Supplementary material for: Marek’s disease virus prolongs survival of primary chicken B-cells by inducing a senescence-like phenotype
Source: PLoS Pathog. 2021 Oct 21;17(10):e1010006. doi: 10.1371/journal.ppat.1010006 (PMC8562793; doi:10.1371/journal.ppat.1010006)
Supplement: Attachment — Submitted filename: Response to reviewers.pdf [file ppat.1010006.s008.pdf]

PPATHOGENS-D-21-00364_Response to Reviewers Comments

Marek's disease virus prolongs survival of primary chicken B-cells by inducing a senescence-like phenotype

Point-to-point responses to reviewers' comments.

Reviewer #1:

In this report by Trapp-Fragnet and colleagues, they use their established in vitro Marek's disease virus (MDV) infection model to study B cell infection. Although not perfect and artificial, this method allows them to study replication in primary B cells and perform many downstream events. Here they examine how MDV affects B cell survival, proliferation, autophagy, and senescence, among other things. First they found that MDV-infected B cells survived longer than uninfected B cells in culture. Through an exhaustive analysis, they systematically examined multiple pathways that could explain the survivability of MDV-infected B cells and found senescence was key to this phenomenon. Contrary to original thinking that MDV induced atrophy in lymphoid organs, such as the bursa, was due to apoptosis in B cells infected with MDV, they found that B cells infected with MDV were actually protected from apoptosis and survived longer than uninfected B cells. This was a well-written manuscript, but a few issues should be addressed.

We thank the reviewer for the thorough review and these positive comments.

1) *The first experiment suggesting B cell viability is confusing. The authors use the term "control" for multiple groups in the results, materials and methods, and figure legend that makes it confusing as to what they are comparing. For example, the text (line 104) says bursal cells treated with CD40L but not with CEC is a control. Control for what and is this the control in Figure 1? If so, this is not the proper control for this experiment. I surmised the "control" in Figure 1 are viable cells collected by FACs that were not GFP positive, as this would be a proper control, but it is not clear in the text.*

Thank you for this important comment. We agree with the reviewer that description and presentation of experimental controls in the original manuscript was insufficient and have addressed this issue as follows:

Actually, all experiments were done with two control groups: i) FACS-purified viable, GFP negative B cells from the infected cultures and ii) uninfected B cells cultured separately. This latter uninfected control group was chosen to exclude the potential effects of infected cells on uninfected cells and a potential "contamination" of the GFP negative control group with cells infected with UL47-GFP MDV at an early stage of infection (when GFP is still not detectable). As both control groups resulted in a similar phenotype in the viability assays, we initially showed only the uninfected control. Now, we included both controls in Figure 1 and clarified this aspect in the respective text (lines 106-119; 206; 440-441; 454-458; 890-893).

2) "*Measuring the total number of cells following incubation with uninfected and infected cells is flawed (Fig. 1). It is not clear if the viable non-GFP and GFP cells obtained by FACS started at the same number of cells. It is mentioned for RNA samples, 3 million cells were used, but it is not clear if this same sample preparation was used for Figure 1. Showing the total number of viable cells at 48 and 72 hours is not justified as the total number of viable cells at days 0*

(after incubation on CECs) and 24 hours should also be shown. Unless the total number of cells at day 0 was same for both groups. It would be more logical to represent the data as the % viable over time (if the numbers were different) since the number of viable cells is most likely different from the start. This should be either shown in the data or represented better."

Thank you for pointing this out. We apologize for the unclear description of our experimental procedure. For the survival assay, infected and control cultures started with the identical amount of cells (0h). After 24h the respective cell populations were sort-purified from these cultures (viable GFP positive and GFP negative B cells form infected cultures and viable B cells from uninfected cultures). These sort purified cells were seeded with equal numbers of $2x10^5$ cells/96-well and cultured for additional 24 (=48h post infection) or 48 h (= 72h post infection) before the number of viable cells was determined. As recommended by the reviewer, we now clarified this in material and methods and provide exact cell counts that were analyzed in each assay (lines 110-111; 478-480; 502; 513; 520; 531).

3) *The data on autophagy is not convincing (Fig 6; S2). To verify the L3B antibody works on chicken cells and is a marker for autophagy, the authors need to use a chemical inducer of autophagy as a control. Likewise, EM of potential autophagosomes is not convincing as the image shown is not clear. Even when zooming in, it is difficult to see the double-walled autophagosomes and using a stress inducing agent as a control would help in this regard.*

As suggested by the reviewer, we provide additional data from new experiments and now included a positive control of autophagy induction using rapamycin in revised Fig.6 (B and C) (lines 246-252; 541-544; 945-948). These data show that Lc3-II is indeed detected in avian cells after induction of autophagy, and confirm that MDV infection of B-cells does trigger autophagy in these cells.

We also agree with the reviewer that demonstration of autophagosomes in the EM image was not convincing and hence, have removed it from the manuscript.

Reviewer #2:

Using cultivation of B cells, FACS sorting, and Microarrays, the authors show that infected B cells are in a state of senescence, which might allow the virus more time to replicate or more time to infect T-cells. All this work was done using their B cell model, so the contribution of the other cells in the infected Bursa was excluded but likely important in deciphering what actually is happening in birds. They eloquently connected their microarray data with FACS analysis to determine what biological pathways are most likely affected in the infected B cells. Other researchers usually show what genes go up and down without making any connection to a biological function, not this group! Frankly really liked this manuscript, although I would like to see more modern techniques being used (i.e., the single-cell transcriptome of cells in the bursa). An atlas of MDV infected Bursa, so the speak.

We thank the reviewer for his enthusiasm concerning our study and his recommendation to use more modern techniques. This will indeed be the next step as we are planning to explore the cellular response of bursal B-cells to MDV infection in future studies by using single-cell resolution approaches.

Reviewer #3:

In this manuscript, Trapp-Fragnet et al. explore the impact of Marek's Disease Virus (MDV) on cell survival in vitro. They utilize a co-culture system, previously developed by the lab, to infect primary chicken bursal cells with a particularly productive MDV strain. Having already established an in vitro model of MDV infection, in this manuscript, the authors characterize the phenotypic impact of MDV on host cells during the initial stages of infection. The authors use microarrays, followed by pathway analysis, to compare gene expression between infected and non-infected cells. They find that MDV-infected B cells outlive non-infected controls in vitro, demonstrating that while fewer infected cells proliferate, they are also significantly less apoptotic than their non-infected controls.

We thank the reviewer for the thorough review of our manuscript, which we happily improved based on the reviewer's suggestions.

1) The authors establish infection by culturing bursal cells with infected chicken embryo cells, then purifying infected B-cells via FACS. However, their uninfected control bursal cells were never incubated with chicken embryo cells, leaving open the possibility that the observed differences in survival, apoptosis, cell cycle progression, and gene expression were impacted, at least in part, by one or more factors secreted by the chicken embryo cells. To account for this possibility, a more suitable control may be to co-culture uninfected chicken embryo cells with bursal cells.

Thank you for bringing up this important point. In a first series of experiments from our initial study, we indeed addressed whether the co-culture of uninfected CECs with B-cells could influence B-cell proliferation and cell cycle. As show below, we did not observe differences in B-cell proliferation in BrdU assays and cell cycle analyses. Consequently, we performed our studies with uninfected B-cells cultured without CEC as negative controls as clarified in lines 440-441. But we agree with the reviewer that this might not give the whole picture and hence we included an additional control group in Fig. 1, representing GFP negative (not-infected) cells from infected, CEF containing cultures. Here, we also observed a comparable phenotype as in bursal B-cells cultured in the absence of CECs.

BrDU proliferation assay

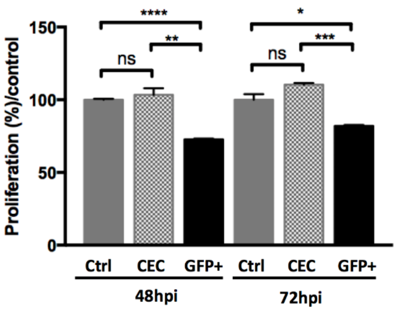

Ctrl : Uninfected B-cells
CEC : Uninfected B-cells co-cultured with uninfected CEC
GFP+ : infected B-cells sorted from co-cultures with MDV infected CEC

Cell cycle analysis

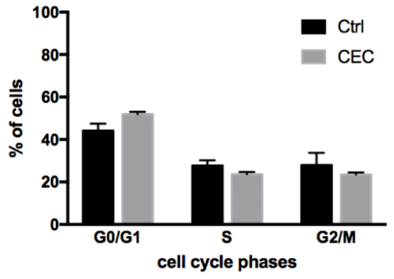

2) *Based on apoptosis and cell cycle analyses, the authors conclude that MDV-infected cells escape cell death by undergoing senescence. This hypothesis should be directly tested using B-gal staining or another established senescent cell detection system. It may be possible that MDV-infected cells delay apoptosis by a day or two without ever truly transitioning to long-term senescence.*

Thank you very much for pointing this out. In the present study, we strived to perform biological tests in order to confirm the microarray results. In this context, we also attempted to develop the senescence test mentioned by the reviewer. However, despite repeated efforts to establish this test based on the measurement of SA-β-gal activity in avian B-cells, we have not been successful. The lack of tools and methods is often a drawback when studying chicken lymphocytes as they exhibit peculiarities compared to mammalian lymphocytes. We agree with the reviewer that due to the lack of a conclusive assay we cannot definitely state that MDV infected B-cells undergo senescence, but we are convinced, our overall results support the view that infected B-cells exhibit characteristics of senescent cells. Therefore, we have chosen the term "senescence-like phenotype" in the manuscript instead of “senescence” to reflect our data.

3) *This study lacks mechanistic detail and much of the data is descriptive. It is important to understand how viral infection increases host cell viability.*

We absolutely agree with the reviewer that the exploration of the mechanisms leading to B-cell survival triggered by MDV is extremely interesting. On the cellular side, we tried to support all transcriptomic data with functional assays (proliferation, apoptosis, autophagy). A first attempt towards the mechanistic question on viral side was the examination of a Meq-deficient virus, which demonstrated the anti-apoptotic oncoprotein is not involved in the observed phenomenon. But clearly, further in depth-studies are needed and will be performed in the future to elucidate these mechanisms more precisely. We know that at this point of our study, we can only formulate well-educated speculations and extrapolations of our data and further emphasized that in the discussion of the revised version of our manuscript (line 379-397).

Minor Issues

Reviewer #1:

1) In Figure 5b, is the Meq-null virus derived from the control (RB1B-GFP) that has pUL47 tagged? Or is it derived from the TK-GFP virus? In other words, is it being compared to its parent virus?

Thank you for this question. The Meq-null virus in Fig. 5b is derived from the TK-GFP virus and has been compared to its parental virus (RB-1B_pTK-GFP). This is now stated in methods (442-445) and the figure legend (933-934).

2) Line 50; MDV is Gallid alphaherpesvirus 2.

3) Line 113; “contrast”

4) Line 312; “cytokines”

Reviewer #2:

There are no major or so that, no minor issues, just the use of the word "precise" as a verb. This was odd. More commonly it is used as "to more precisely define."

Also using "for the first time" is unnecessary.

In this sentence, "Though on first glance a peculiar feature, the senescence-like phenotype of bursal leukocytes could promote MDV life cycle and/or the persistence of the virus thus allowing it to have more time to recruit and infect T-cells," delete "MDV life cycle and/or." The persistence of MDV is part of the life cycle.

Thanks you for these comments, which we all implemented in the manuscript as suggested :

- line 193; “more precisely define”
- lines 377 and 419; “for the first time” has been removed
  line 410; "MDV life cycle and/or." has been deleted

Reviewer #3:

1) *Autophagy results in Fig 6B should be more quantitative (maybe by counting cells with and without marker), and include statistics.*

As recommended, we counted B-cells showing the Lc3-II staining to quantify the proportion of cells in autophagy. These data are now shown in Fig. 6C in the revised version of the manuscript and presented in the text (lines 246-252; 554-556 and 948-949). Thanks for this suggestion that indeed strengthen our results.

2) *The conclusion stated in the subheader, “MDV infections strongly regulate gene expression in B-cells” (line 114), is…*

…that MDV infection triggers massive changes in the abundance of mRNA transcripts in B-cells that could have an impact on B-cell survival at different levels.

3) *Microarray data in Figs 2A, 3A, 4A, 6A, and 7A should indicate significance and include error bars. Or apply rank-order statistical tests on all genes of a given pathway (i.e. KS test or similar). Somehow the variation should be taken into consideration.*

Thank you for this comment. In order to show the variation of the data we included the adjusted p-values for all pictured genes into supplemental tables and clarified this point in the methods section (489-497).

4) *Y-axis of microarray data should indicate that it is log(fold change) not just fold change. I assume log because there are negative values – y-axis should indicate the appropriate calculation.*

As rightfully pointed out by the reviewer, the description in the method section for microarray data was incomplete. We do indeed show FC values but for an easier understanding of gene downregulation, FC values below 1 were converted into negative values according to the formula [-1/FC]. This has now been added to the method section together with a more detailed explanation of the FC calculation (line 489-497).
