# Supplementary material for: Marek’s disease virus prolongs survival of primary chicken B-cells by inducing a senescence-like phenotype
Source: PLoS Pathog. 2021 Oct 21;17(10):e1010006. doi: 10.1371/journal.ppat.1010006 (PMC8562793; doi:10.1371/journal.ppat.1010006)
Supplement: Attachment — Submitted filename: response to reviewers.pdf [file ppat.1010006.s009.pdf]

PPATHOGENS-D-21-00364_Response to Reviewers Comments

# Marek's disease virus prolongs survival of primary chicken B-cells by inducing a senescence-like phenotype

**Reviewer 1:**

We would like to thank the reviewer #1 for his comments, which greatly improved our initial submission, and we are happy that all of the revisions have met his expectations.

**Reviewer 3 and Editor:**

Reviewer #3 raised a final question on the role of CEC on uninfected bursal B-cells. The Editor also recommended including the appropriate control for our microarray analysis.
In our microarray, we also sorted uninfected B-cells (GFP-negative) from the infected B-cell cultures. These B-cells were present in the same milieu, were in contact with CEC, and are an optimal control. To address the reviewer's questions, we performed an in-depth analysis of the microarray dataset including this GFP-negative population and included this new data in the manuscript (line 499-503 and Table S1). The comparison of the data obtained from GFP-negative (uninfected) B-cells co-cultivated with infected CEC and uninfected B-cells cultivated alone (control initially shown in our study), revealed that overall more than 81% of the genes analyzed are expressed similarly between the two groups. This analysis therefore suggests that CEC have a very limited effect on B-cells. Of note, the differentially expressed genes are mostly IFN-stimulated genes (e.g. Mx1, PKR, IFIT5), as expected for co-culture conditions.

To attend to reviewer #3 concerns regarding the role of CEC in regulating autophagy and apoptosis pathways in B-cells specifically, we also examined DEGs in GFP-positive (infected) cells compared to GFP-negative (uninfected) B-cells from the same culture or versus the CEC free control cells. As shown below, the expression of genes involved in autophagy and apoptosis showed a very good agreement with R-squared values of 0.82 and 0.94, respectively. These comparisons confirm that those genes are similarly expressed in uninfected cells whether or not they have been co-cultured with CEC.

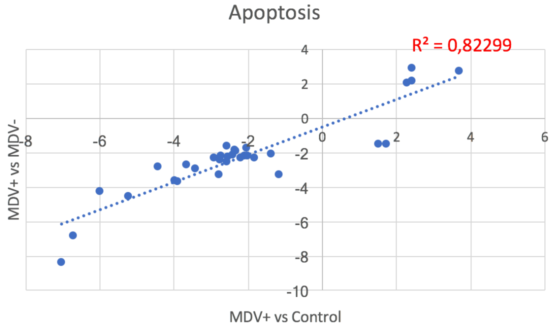


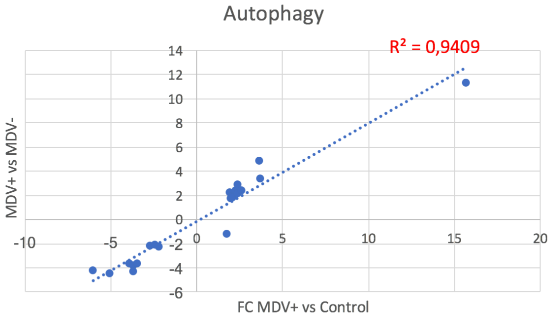


To support our findings that CEC play only very minor roles on B-cell regulations, we also compared our microarray dataset to RNAseq data obtained in a previous study (Bertzbach et al., Viruses, 2019, doi: 10.3390/v11030264). The RNA seq analysis was conducted on B-cells that were (mock-) infected in co-cultures with mock-infected CEC (as suggested by the reviewer) or RB-1B_pTK-GFP-infected CEC. Although FC values differ between the experiments due to different conditions of infection (virus, time and chicken line) and the methodology, we observed a similar trend of changes in the gene expression profiles between our microarray and the previous RNAseq analysis (see below).

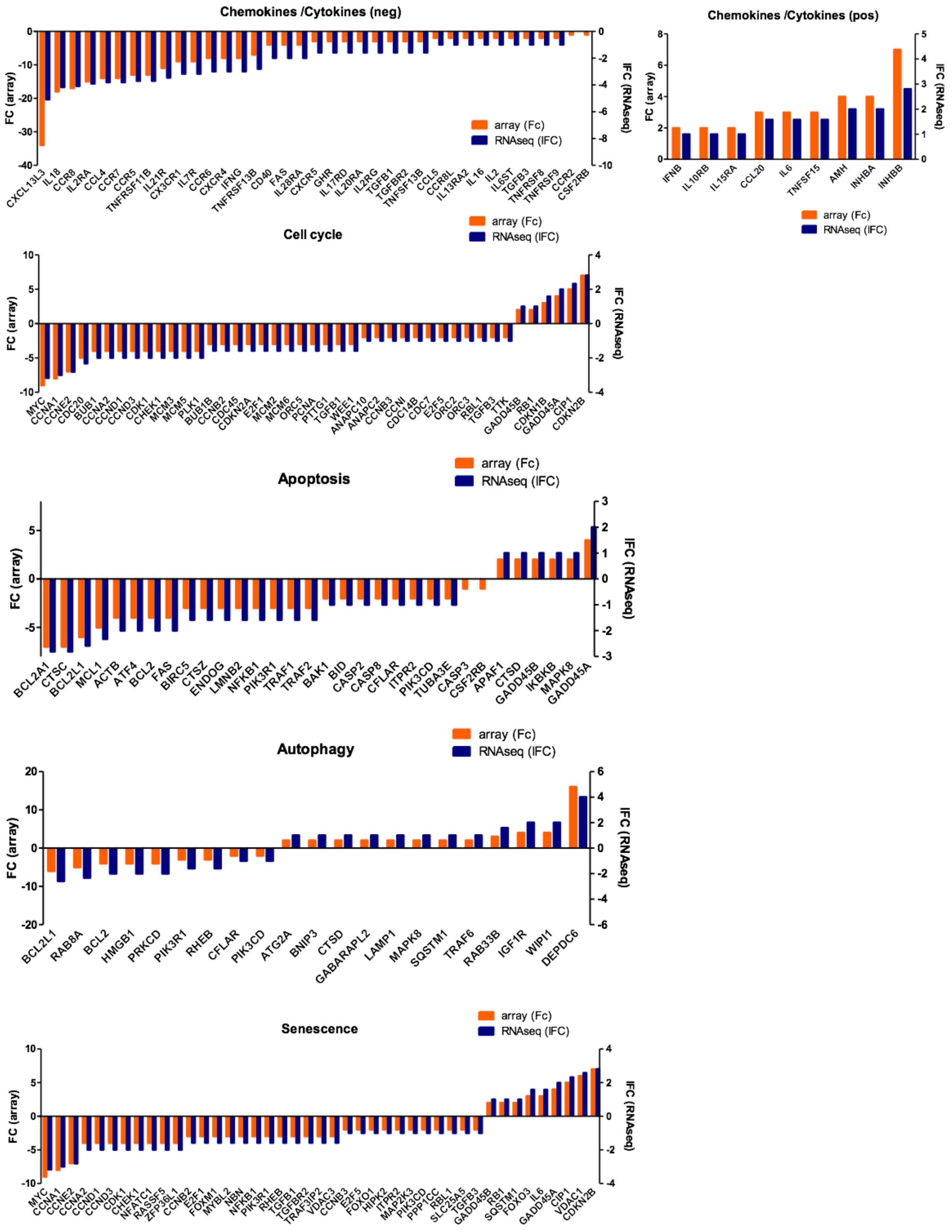


This data comparison reinforces our microarray data and answers the reviewer's question on the effects of CEC on uninfected bursal B-cells. We hope that with the new data and the changes made, the manuscript will be accepted for publication at PLoS Pathogens.
